# Loss, mutation and deregulation of *L3MBTL4 *in breast cancers

**DOI:** 10.1186/1476-4598-9-213

**Published:** 2010-08-10

**Authors:** Lynda Addou-Klouche, José Adélaïde, Pascal Finetti, Nathalie Cervera, Anthony Ferrari, Ismahane Bekhouche, Fabrice Sircoulomb, Christos Sotiriou, Patrice Viens, Soraya Moulessehoul, François Bertucci, Daniel Birnbaum, Max Chaffanet

**Affiliations:** 1 Marseille Cancer Research Center; Department of Molecular Oncology, UMR891 Inserm; Institut Paoli-Calmettes; Marseille, France; 2 Functional genomics and translational research unit, Institut Jules Bordet, Brussels, Belgium; 3 Department of Medical Oncology, Institut Paoli-Calmettes, Marseille, France; 4 Université de la Méditerranée, Marseille, France; 5 Biotoxicology laboratory. Djillali Liabes University, Sidi-Bel-Abbès, Algeria

## Abstract

**Background:**

Many alterations are involved in mammary oncogenesis, including amplifications of oncogenes and losses of tumor suppressor genes (TSG). Losses may affect almost all chromosome arms and many TSGs remain to be identified.

**Results:**

We studied 307 primary breast tumors and 47 breast cancer cell lines by high resolution array comparative genomic hybridization (aCGH). We identified a region on 18p11.31 lost in about 20% of the tumors and 40% of the cell lines. The minimal common region of loss (Chr18:6,366,938-6,375,929 bp) targeted the *L3MBTL4 *gene. This gene was also targeted by breakage in one tumor and in two cell lines. We studied the exon sequence of *L3MBTL4 *in 180 primary tumor samples and 47 cell lines and found six missense and one nonsense heterozygous mutations. Compared with normal breast tissue, *L3MBTL4 *mRNA expression was downregulated in 73% of the tumors notably in luminal, ERBB2 and normal-like subtypes. Losses of the 18p11 region were associated with low *L3MBTL4 *expression level. Integrated analysis combining genome and gene expression profiles of the same tumors pointed to 14 other potential 18p TSG candidates. Downregulated expression of *ZFP161, PPP4R1 *and *YES1 *was correlated with luminal B molecular subtype. Low *ZFP161 *gene expression was associated with adverse clinical outcome.

**Conclusion:**

We have identified *L3MBTL4 *as a potential TSG of chromosome arm 18p. The gene is targeted by deletion, breakage and mutations and its mRNA is downregulated in breast tumors. Additional 18p TSG candidates might explain the aggressive phenotype associated with the loss of 18p in breast tumors.

## Background

The development and progression of breast cancer is the result of the accumulation of genetic alterations such as amplification of oncogenes and deletions of tumor suppressor genes (TSG) in the epithelial cells of the mammary gland. Frequent deletions have been reported on chromosome arms 1p, 3p, 7q, 8p 9p, 16q and 17p but only few reports describe such deletions on chromosome arm 18p [1-3].

We profiled a series of 307 primary breast tumors and 47 breast cancer cell lines by using high resolution array comparative genomic hybridization (aCGH). We identified a region on 18p11.31 deleted in 25% of the tumors and 40% of the cell lines. We delineated a minimal common region of deletion that targeted the *L3MBTL4 *gene. The *L3MBTL4 *gene is one of the four human orthologs of *Drosophila *lethal (3) malignant brain tumor (*l(3)mbt*). *L(3)mbt *is a *bona fide *TSG in the fly [4,5]. The L3MBTL4 protein contains three "malignant brain tumor" (MBT) domains. This domain of about 100 amino acid residues is conserved in protostomians and deuterostomians and often exists as repeats [6]. The MBT domain binds methylated histone residues. The human genome contains several MBT-containing proteins, some of which have been linked to gene regulatory pathways and polycomb-mediated repression, and to cancer [7]. *L3MBTL1 *is a TSG implicated in myeloid malignancies [8], and *L3MBTL3 *deregulation is associated with neuroblastoma [9].

To document the involvement of *L3MBTL4 *in breast cancer we searched for mutations by sequence analysis of 180 primary tumor samples and 47 cell lines. We found that, in addition to deletions, *L3MBTL4 *is targeted by mutations. Finally, we found that *L3MBTL4 *deletions correlate with low mRNA expression and with the presence of lymph node metastasis, high Scarf-Bloom-Richardson (SBR) grade and luminal B molecular subtype.

Our study is the first to identify a region on chromosome arm 18p likely to contain a putative TSG involved in breast oncogenesis, and to show that this TSG may be *L3MBTL4*. We did not exclude the existence of other potential 18p TSGs, some of which could explain the aggressive phenotype of breast tumors with 18p loss.

## Samples and Methods

### Breast tumors

Tumor tissues were collected from 307 patients with primary adenocarcinoma who underwent initial surgery at the Institut Paoli-Calmettes (Marseille, France) between 1992 and 2004. Immediately after macroscopic examination of the surgery specimen by two pathologists, samples containing more than 60% of tumor cells were obtained, frozen in liquid nitrogen and stored at -80°C until nucleic acids extraction. The main histoclinical characteristics of tumors are listed in the Additional file 1, Table S1. Each patient gave written informed consent and the study was approved by our institutional review committee.

### Cell lines

The 47 breast cell lines used in this study were BT-20, BT-483, HCC1937, CAMA-1, HCC38, HCC1500 [10], Hs 578T [11], BT-474, BT-549, HCC202, HCC1395, HCC1569, HCC1806, HCC1954, HCC2218, HME-1, MCF7, MDA-MB-134, MDA-MB-157, MDA-MB-175, MDA-MB-231, MDA-MB-361, MDA-MB-415, MDA-MB-436, MDA-MB-453, MDA-MB-468, SK-BR-3, SK-BR-7, T47 D, UACC-812, UACC-893, ZR-75-1, ZR-75-30 (ATTC, Manassas, VA), BrCa-Mz-01, BrCa-Mz-02 [12], SUM44, SUM52, SUM102, SUM149, SUM159, SUM185, SUM190, SUM206, SUM225, SUM229 [13], S68 (V. Catros, Rennes, France), and CAL51 [14]. All cell lines were grown as recommended by their suppliers.

### DNA and RNA extraction

DNA and RNA were extracted from frozen samples by using guanidium isothiocynanate and cesium chloride gradient, as previously described [15]. DNA quality and RNA integrity were respectively controled on polyacrylamide gel electrophoresis and on Agilent Bioanalyzer (Agilent Technologies, Massy, France). DNA was also extracted from a normal area of paraffin-embedded T8584, T8847, T9193 and T8525 tissues.

### Genome analysis by array-comparative genomic hybridization (aCGH)

Genome profiles were established on 307 tumors and 47 breast cancer cell lines by aCGH using 244K CGH Microarrays (Hu-244A, Agilent Technologies, Massy, France) as previously described [16]. A pool of 13 normal male DNAs was used as reference. Scanning was done with Agilent Autofocus Dynamic Scanner (G2565BA, Agilent Technologies). Data analysis was done and visualized with CGH Analytics 3.4 software (Agilent Technologies). Extraction of data (log_2 _ratio) was done from CGH analytics, while normalized and filtered log_2 _ratio were obtained from "Feature extraction" software (Agilent Technologies). Data generated by probes mapped to × and Y chromosomes were eliminated. The final dataset contained 225,388 unique probes covering 22,509 genes and intergenic regions according to the hg17/NCBI human genome mapping database (build 35). Data were analyzed using circular binary segmentation (CBS) [17] as implemented in the DNA copy R/Bioconductor package [18] with default parameters to translate intensity measurements in regions of equal copy number, each region being defined by at least five consecutive probes. Thus, each probe was assigned a segment value referred to as its "smoothed" value.

We used a threshold value of |0.33| (log_2 _ratio) to define a copy number aberration (CNA) [16]. Identification of copy number variations (CNV) was done using the regions published by McCarroll et al [19] which are stored in the Database of Genomic Variants (release v8) [20]. To determine altered regions, we used the GISTIC algorithm [21], which computes for each genomic segment through the whole genome a score based on the frequency of CNA combined with its amplitude, with bootstrapping to calculate the significance level (p < 0.001). To establish significant association between CNA and categorical variables, Fisher's exact test was used. Gains and losses were handled separately.

### Mutation analysis

Sequence analysis was done on 180 out of the 307 breast tumors and on the 47 breast cell lines after amplification of genomic DNA. Seventeen primers pairs were designed to amplify *L3MBTL4 *DNA by polymerase chain reaction (PCR) (Additional file 1, Table S2). PCR amplifications were done in a total volume of 25 μl PCR mix containing at least 10 ng template DNA, Taq buffer, 500 μmol of each deoxynucleotide triphosphate, 100 μmol of each primer and 1 unit of Hot Star Taq DNA polymerase (Qiagen, Courtaboeuf, France). PCR amplification conditions were as follows: 95°C 10 min and 95°C 30 sec, annealing T° 55°C 30 sec, 72°C 45 sec to to 1 min depending on PCR product length, 72°C 10 min, 35 cycles. PCR products were purified using Millipore plate MSNU030 (Millipore SAS, Molsheim, France). Aliquots (1 μl) of the purified PCR products were sequenced using Big Dye terminator v1.1 Cycling Sequencing Kit (Applied Biosystems, Courtaboeuf, France) including the forward or reverse primer. The sequencing products were purified through Multi-Screen-HV 96-well filter plates (Millipore, Billerica, MA, USA) preloaded with Sephadex G-50 (Sigma, St Louis, MO, USA). The reactions were run on the ABI 3130XL Genetic Analyser (Applied Biosystems, Courtaboeuf, France). The sequence data files were analyzed using the phred/phrap/consed software. All mutations were confirmed by a second round of PCR and sequencing reactions in both directions.

### Quantitative real time reverse-transcribed PCR (qRT-PCR)

*L3MBTL4 *gene expression level was analyzed by qRT-PCR on a set of 52 out of the 307 tumors. Two μg of total RNA, treated beforehand with RNase-free DNase (Promega, France), was reverse-transcribed using the SuperScript II RT and 100 ng of randomhexamers (Invitrogen, France). PCR reactions were carried out in a LightCycler 2.0 instrument (Roche, Germany) in a final volume of 20 μl according to the supplier's recommendations using LightCycler FastStart DNA Master ^plus ^SYBR Green I Kit (Roche, Germany). *L3MBTL4 *primers, *L3MBTL4*-F (CTTGGAGCAAGCTGAAGAGG) and *L3MBTL4*-R (TGGAAAGGACTGATCCTTGG) (Sigma-Aldrich, Austria), were designed to anneal to exon 4 and exon 6, respectively. Primers for the control gene, *GUSB*, were *GUSB*-F (GAAAATATGTGGTTGGAGAGCT) and *GUSB-R *(CCGAGTGAAGATCCCCTTTTTA) (Sigma-Aldrich, Austria). The mean threshold cycle (Cp) was calculated for each gene and ΔCp was defined as ΔCp = Cp (*GUSB*) - Cp (*L3MBTL4*). The ΔCp was determined on two to six times for each sample and the mean calculated. The fold ratio of *L3MBTL4 *transcripts was calculated using the equation, fold ratio = 2^ΔCp^. Commercial pools of normal breast RNA (Clontech, Palo Alto, CA) were analyzed and used as control.

### Gene expression profiling with DNA microarrays

A total of 229 tumors studied by aCGH, as well as 4 normal breast tissue samples, were profiled with Affymetrix U133 Plus 2.0 human oligonucleotide microarrays as previously described [22]. Scanning was done with Affymetrix GeneArray scanner. Data were analyzed by 'Robust Multichip Average' (RMA) with the non-parametric quantile algorithm as normalization parameter in R/Bioconductor and associated packages [18]. All probes were mapped based on their EntrezGeneID. When multiple probes were mapped to the same gene, the probe sets with an extension « at », next « s_at », and followed by all other extensions were preferentially kept. When several probe sets with the best extension were available, the one with the highest median value was retained.

The five molecular subtypes related to the intrinsic breast cancer tumor classification were determined using the single sample predictor (SSP) classifier [23] associated to 'Distance Weighted Discrimination' (DWD) as data set adjustment [24].

Prior statistical analyses *L3MBTL4 *expression level in samples was centered using its expression level in the 4 normal breast samples pooled. Over- and under-expression were defined using a two-fold threshold i.e. |1| in the log_2 _transformed data.

### Comparative analyses of genome and expression data

To identify other potential 18p TSGs we compared the degree of CNA-driven RNA downregulation in 229 of the 307 samples by analyzing the genes common to the genome and expression platforms (aCGH Agilent Technologies and Affymetrix) and retained after filtering based on the expression level. Briefly, a potential TSG had to show a lower expression value in a sample with loss than in those without (Student t test) and an underexpression frequency overrepresented in samples with the loss (Fisher's exact test). For both tests significance level was 5% with a false discovery rate (FDR, [25]) less than 1%.

### Statistical analyses

Correlations between L3MBTL4 CNA groups and histoclinical factors were calculated with the Fisher's exact test. Student's *t *test and one-way ANOVA test were used to evaluate association of *L3MBTL4 *gene expression level within histoclinical factors. Overall specific survival (OS) and metastasis-free survival (MFS) curves were estimated using the Kaplan-Meier method and statistical significance of pairwise comparisons was assessed using the log-rank test. OS and MFS follow-up times were measured from the date of diagnosis till death from breast cancer and till the first occurrence of distant metastases, respectively. All statistical tests were two-sided at the 5% level of significance. Statistical analysis was done using the survival package (version 2.30), in the R software (version 2.9.1).

## Results

### Losses and breakages of 18p targets the L3MBTL4 gene at p11.31

**Figure 1:**
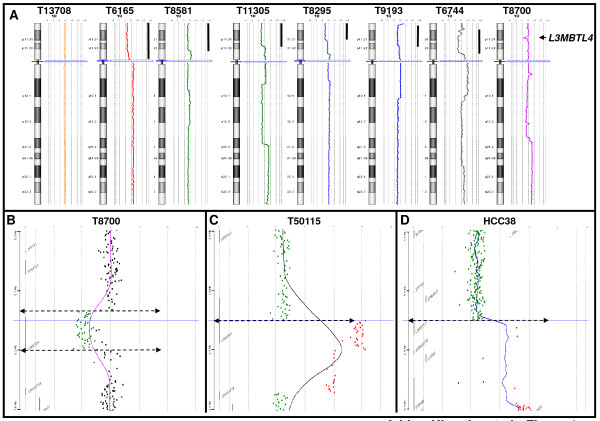
**Examples of chromosome 18 aCGH profiles**. **(A) **Tumor T13708 on the left does not present any gene copy number aberration. Tumors T6165, T8581, T11305, T8295, T9193, T6744 and T8700 exhibit copy number losses on the short arm of chromosome 18. The genomic profile observed in tumor T8700 shows the smallest region targeted by the 18p loss. **(B) **The smallest deletion observed in breast primary tumor T8700 targets the *L3MBTL4 *gene (18p11.31) and includes the minimal 18p common lost region, Chr18:6,366,938-6,375,929 bp defined with 96% of studied tumors (Additional file 1, Table S4A). **(**C, D**) **Breakpoint targeting the *L3MBTL4 *gene in breast primary tumor T50115 and HCC38 breast cancer cell line.

Genome profiles of 307 primary breast tumors and 47 cancer cell lines were established by aCGH. GISTIC analysis showed that chromosome arm 18p was targeted by losses in more than 20% of tumor samples (Additional file 2, Figure S1). None of these losses were homozygous deletions. Profiles showed various sizes of losses (Figure 1A). In tumor T8700 loss within 18p11.31 (Figure 1A) spanned the *L3MBTL4 *gene only (Figure 1B). 18p losses including this region were found in 77 (25%) primary tumors and 19 (40%) cell lines.

Copy number transitions are likely to reflect DNA strand breakages that may lead to nonreciprocal translocations [26]. *L3MBTL4 *was targeted by copy number transition in tumor T50115, and in the HCC38 (Figure 1C, D) and MDA-MB-453 cell lines. In T50115, the breakpoint was within the genome interval [ch18: 6,202,799-6,212,615], which contains *L3MBTL4 *exons 10 and 11. The resulting protein should be truncated of its C-terminal part starting from the second MBT motif. In HCC38 and MDA-MB-453 the breakpoints spanned genome intervals chr18: 6,009,803-6,020,061, (between exons 17 and 18) and chr18: 6,030,519-6,044,093 (including exon 16], respectively. These two breakages should generate a protein truncated of the sterile alpha motif (SAM) domain. None of these potential fusion transcripts involving the *L3MBTL4 *gene has been identified yet.

Thus, the *L3MBTL4 *gene is targeted by various genomic alterations, including loss and breakage, in a high proportion of breast cancers.

### Features of tumors with L3MBTL4 loss

*L3MBTL1*, *L3MBTL2 *and *L3MBTL3 *paralogs were not targeted by deletion in our breast tumor set. We did not find any *L3MBTL4 *deletion among genome profiles similarly established in 80 colon cancers, 115 myeloid hematopoietic diseases and 53 sarcomas (data not shown). This suggests that *L3MBTL4 *loss occurs specifically in breast cancers.

Comparison of aCGH data in tumors with and without *L3MBTL4 *loss showed that *L3MBTL4 *loss was often found together with the loss of four 17q genes: *ACCN1 *(p = 2.5.10^-5^), *RHOT1 *(p = 8.3.10^-5^), *PSMD11 *(p = 1.3.10^-4^), and *MYO1 D *(p = 1.8.10^-4^) (Additional file 1, Table S3).

The 77 tumors with *L3MBTL4 *loss comprised 18 luminal A, 27 luminal B, 17 basal, 9 ERBB2, 2 normal-like (Table 1) and 4 non-informed tumors (Additional file 1, Table S4A). Among these, 42 (55%) showed loss of a regional part of chromosome 18p including the *L3MBTL4 *gene. They included 8 luminal A, 15 luminal B, 10 basal, 5 ERBB2, 1 normal-like and 3 non-informed tumors. The minimal commonly deleted region (CDR) including *L3MBTL4 *was defined for each molecular subtype as chr18:6,366,938-6,798,785 bp in luminal A (telomeric and centromeric limits were defined by tumors T11348 and T8683, respectively and noted [T11348-T8683]), chr18:3,784,366-6,202,799 bp in luminal B ([T9377-T50115]), chr18:6,285,396-6,375,929 bp in basal ([T5142cc-T8700]), chr18:3,732,722-6,168,500 bp in ERBB2 ([T9296]), chr18:5,520,302-8,628,361 bp in normal-like ([T12652]) and chr18:5,200,158-11,854,021 bp in non-informed tumors ([T6744-T12662]) (Additional file 1, Table S4A).

**Table 1:** Comparison of clinical features between breast tumors associated or not with *L3MBTL4 *loss (determined by aCGH)

Characteristics (N)	*L3MBTL4 *non deleted	*L3MBTL4 *deleted	p	odds ratio
	N = 230	N = 77		
Age (300)	52 (22-84)	51 (24-82)	0.69	
Histological type (255)			0.57	
Ductal	152 (78%)	48 (79%)		
Lobular	15 (8%)	5 (8%)		
Medularry	12 (6%)	2 (3%)		
Mixt	8 (4%)	3 (5%)		
Other	7(4%)	3 (5%)		
Clinical form (301)			0.11	1.76
IBC	33 (15%)	18 (23%)		(0.87-3.5)
Non IBC	191 (85%)	59 (77%)		
Pathological tumor size (240)			0.052	
pT1	45 (25%)	8 (14%)		
pT2	95 (52%)	28 (48%)		
pT3	42 (23%)	22 (38%)		
Pathological axillary lymph node status (266)			**1.02E-02**	0.46
Negative	96 (48%)	20 (30%)		(0.24-0.85)
Positive	103 (52%)	47 (70%)		
SBR grade (267)			**1.15E-02**	
1	38 (19%)	3 (5%)		
2	63 (31%)	25 (38%)		
3	100 (50%)	38 (58%)		
ER (275)			0.11	0.6
Negative	84 (40%)	19 (29%)		(0.31-1.13)
Positive	125 (60%)	47 (71%)		
PR (264)			0.32	0.74
Negative	96 (48%)	26 (41%)		(0.4-1.36)
Positive	104 (52%)	38 (59%)		
P53 (192)			0.39	1.38
Negative	87 (59%)	30 (67%)		(0.65-3)
Positive	60 (41%)	15 (33%)		
Ki67 (217)			0.23	0.64
Negative	53 (32%)	12 (23%)		(0.28-1.36)
Positive	112 (68%)	40 (77%)		
ERBB2 (226)			1	1.03
Negative	145 (85%)	48 (86%)		(0.42-2.83)
Positive	25 (15%)	8 (14%)		
Molecular subtype (264)			**8.04E-04**	
Basal	63 (33%)	17 (23%)		
ERBB2	22 (12%)	9 (12%)		
Luminal A	52 (27%)	18 (25%)		
Luminal B	29 (15%)	27 (37%)		
Normal-like	25 (13%)	2 (3%)		
Metastatic relapse (198)			0.32	0.62
no	135 (88%)	37 (82%)		(0.23-1.78)
yes	18 (12%)	8 (18%)		
Death from breast cancer (239)			0.38	0.73
no	139 (77%)	41 (71%)		(0.36-1.52)
yes	42 (23%)	17 (29%)		
5 year-Metastasis-free survival (198)	90%	81%	0.254	
5 year-Overall specific survival (239)	78%	73%	0.271	

Note: IBC, Inflammatory Breast Cancer; SBR, Scarf-Bloom-Richardson, histological grade.

The 35 other cases showed a loss of *L3MBTL4 *as a consequence of the complete loss of the chromosome 18p. They included 10 luminal A, 12 luminal B, 7 basal, 4 ERBB2, 1 normal-like and 1 non-informed tumors. Interestingly, the complete loss of the chromosome 18p was associated with the luminal B molecular subtype (p < 0.05), as well as cancer in older women (>50 years old) (Additional file 1, Table S4B).

Taken together, a minimal 18p CDR, chr18:6,366,938-6,375,929 bp within the *L3MBTL4 *locus, was defined in 74 tumors (Additional file 1, Table S4A). In T9296, T50115 and T50136 the *L3MBTL4 *loss flanked this CDR at the telomeric and centromeric borders, respectively.

Comparison of clinical features between breast tumors with and without *L3MBTL4 *loss (Table 1) showed that *L3MBTL4 *loss was associated with the presence of lymph node metastases (p = 1.02 10^-2^), high SBR grade (p = 1.15 10^-2^) and luminal B molecular subtype defined by SSP classification [23] (p = 3.10^-5^). No impact on survival was noted.

### L3MBTL4 is targeted by mutations

**Figure 2:**
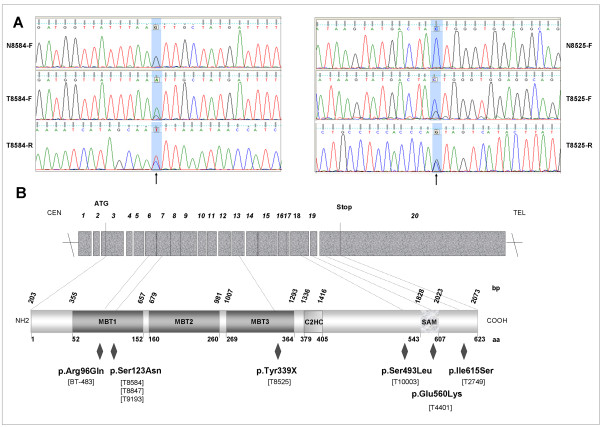
**Mutations of *L3MBTL4 *gene in 180 breast tumors and 47 breast cancer cell lines**. **(A) **Examples of mutations in the *L3MBTL4 *gene. Sequence detects a c.368G>A mutation in exon 7 in T8584. Mutation c.1017C>G in exon 13 generates a stop codon in T8525. The absences of the corresponding mutation in paired normal tissues (N8584 and N8525) suggest that the mutations were acquired. Vertical arrows indicate the position of the mutation. **(B) **Representation of L3MBTL4 protein and localization of the mutations. For each tumor, mutations were identified by sequence analysis and translated on the L3MBTL4 protein (lozenges). Primers and conditions used are described in the Additional file 1, Table S2.

We searched for mutation in *L3MBTL4 *exons in 180 and 47 of the aCGH-profiled tumors and cell lines, respectively. Sequence analysis of the tumor samples identified 32 variants including 25 synonymous (23 p.Ileu570Ileu, 2 p.Val201Val), 6 missense (3 p.Ser123Asn, 1 p.Ser493Leu, 1 p.Glu560Lys, 1 p.Ile615Ser) and 1 nonsense (p.Tyr339X). In BT-483 cell line a missense (p.Arg96Gln) mutation was identified. Figure 2A shows two examples of mutation. The localization and nature of the mutations are shown in Figure 2B. The p.Ser123Asn substitution and the nonsense mutation were located in regions encoding the conserved MBT1 and MBT3 motifs, respectively. The nonsense mutation should generate a truncated L3MBTL4 protein without C2HC zinc finger and SAM domains. The p.Arg96Gln substitution in BT-483 should affect the MBT1 domain. The SAM motif was also targeted by the p.Glu560Lys substitution. We were able to show that the p.Ser123Asn and p.Tyr339X mutations found respectively in T8584, T8847 and T9193 and in T8525, were acquired (Figure 2A). We could not confirm that the other missense mutations are similarly somatic. However, they were not included as SNP or missenses in NCBI db SNP build 131 [27].

Counting only the 7 potentially deleterious mutations the frequency of such event would be 3.9% in tumor samples.

Two mutated (missense mutation) tumors (T9193 and T10003) were also deleted.

### L3MBTL4 mRNA is downregulated in breast tumors

**Figure 3:**
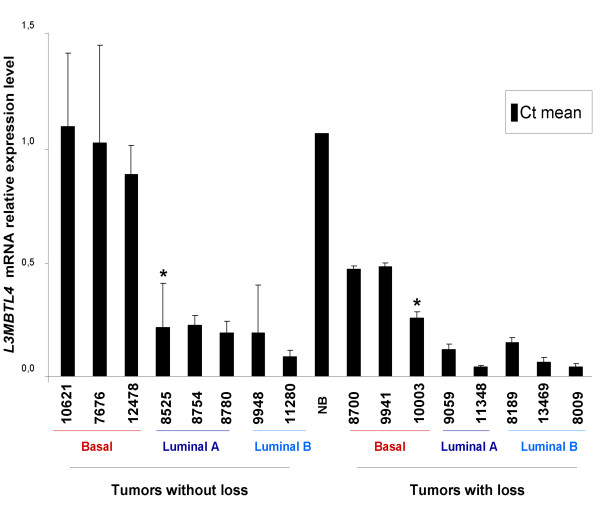
**Comparison of *L3MBTL4 *mRNA levels in deleted and non deleted breast tumors**. mRNA levels were measured by quantitative RT-PCR. *GUSB *mRNA expression was used as internal control for mRNA normalization. *L3MBTL4 *mRNA expression in deleted tumors was lower than in normal breast (NB). Among the non-deleted tumors, *L3MBTL4 *mRNA expression was lower in luminal than in normal breast (NB) and basal tumors. This suggests that the *L3MBTL4 *gene expression can be affected by heterozygous deletion but also regulated by an epigenetic mechanism. Asterisk (*) indicates the mutated samples.

*L3MBTL4 *gene expression was measured using qRT-PCR in normal breast tissues and 52 breast tumor samples including 16 with a loss of the *L3MBTL4 *gene region (T7420, T8009, T8189, T8600, T8700, T9059, T9398, T9888, T9941, T11348, T11485, T11568, T10684, T12854, T13469, T13018), one mutated and deleted (T10003), and one mutated but not deleted (T8525). *L3MBTL4 *gene expression was quantified by comparison with the expression of the housekeeping *GUS *gene. Overall, tumor samples expressed a low level of *L3MBTL4 *mRNA as compared with normal breast tissue. *L3MBTL4 *mRNA level was decreased at least two-fold in all tumors with *L3MBTL4 *loss as well as in the mutated and non-deleted sample (Figure 3). Some non-deleted tumors also exhibited low *L3MBTL4 *mRNA level suggesting that *L3MBTL4 *downregulation could be attributed to several mechanisms including deletions but also regulation of transcription.

**Figure 4:**
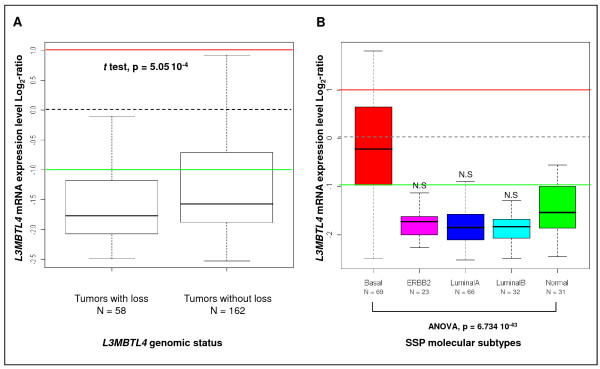
**Boxplots representing *L3MBTL4 *mRNA expression level in breast tumors**. **(A) **Comparison of *L3MBTL4 *expression according to its genomic status. *L3MBTL4 *expression level (log_2 _ratio) was lower in tumors with than without deletion (p = 5.05^e-4^). Red and green horizontal lines indicate thresholds (i.e. Basal tumors exhibited a normal mRNA expression (Figure 4B). However, the correlation between *L3MBTL4 *mRNA downregulation and gene loss was not simply a consequence of the proportion of basal tumors in the category "tumors without loss". Indeed, a similar proportion of basal tumors [26% (15/58) and 32% (52/162)] was observed in both "tumors with loss" and "tumors without loss" groups, respectively (Fisher test, p = 0.41). Moreover, downregulated *L3MBTL4 *expression was correlated with *L3MBTL4 *gene loss in basal tumors (p < 0.05).

We also determined *L3MBTL4 *gene expression in 229 tumor samples and 4 NB samples by using Affymetrix microarrays. *L3MBTL4 *mRNA was downregulated (ratio T/NB <0.5) in 166 tumors (72%). Comparative analysis of these 229 samples identified a significant correlation (p = 5.05 10^-4^) between *L3MBTL4 *mRNA downregulation and gene loss (Figure 4A). Downregulation of *L3MBTL4 *mRNA was associated with luminal A, luminal B and ERBB2 molecular subtypes (p = 6.734 10^-43^).

Deletion, mutation and expression data are described in the Additional file 1, Table S4A and summarized in Table 2.

**Table 2:** Summary of *L3MBTL4 *alterations

Samples	Loss	Mutation	Loss and mutation	Breakages	*mRNA downregulation
**Total tumors **(N = 307; *229)	77	7(/180 studied)	2	1	166
Luminal A (N = 70; *66)	18	1	0		62
Luminal B (N = 56; *47)	27	1	0	1(T50115)	45
ERBB2 (N = 31; *23)	9	2	1		22
Basal (N = 80; *67)	17	1	1		16
Normal-like (N = 27; *26)	2	0	0		21
Not informed (N = 43)	4	2	0		0

**Total cell lines **(N = 47)	19	1	1	2	Not applicable
Luminal cell lines(N = 17)	12	1	1 (BT-483)	1 (MDA-MB-453)	Not applicable
Basal cell lines (N = 9)	2	0	0	1 (HCC38)	Not applicable
Others/Not done (N = 21)	5	0	0	0	Not applicable

* Samples analyzed by using DNA microarrays.

Expression microarray profile revealed five genes (*TTMA*, *PTPN2, NDC80, SLMO1 *and *TUBB6*) coordinately deregulated with *L3MBTL4 *and also localized in an 18p region frequently lost in breast tumors, suggesting a common mechanism of deregulation of their expression, which might contribute to breast oncogenesis.

Correlations between *L3MBTL4 *microarray data and clinical features of 229 tumors are reported in the Additional file 1, Table S5. The lowest expression of *L3MBTL4 *was found in lobular tumors (p = 7,11.10^-12^) and in inflammatory breast cancers (p = 0.04) and was associated with the expression of estrogen receptor (ER) (p = 2,73.10^-14^), progesterone receptor (PR) (p = 3,11.10^-13^), ERBB2 (p = 7,73.10^-14^), SBR grade 2 (p = 1.49 10^4^), lymph node metastasis (p = 3,39.10^-5^), P53 negativity (p = 0.0495) and low expression of Ki67 proliferative index (p = 19,61 10^-5^).

### Sixty-four genes are targeted by 18p copy number losses in breast cancer

To identify other 18p genes targeted by copy number losses, a GISTIC analysis was done on the genome profiles of the 307 cases. Recurrent copy number losses targeted 64 genes within the 18p11.21-11.32 region (p < 1 × 10^-5^) (Additional file 2, Figure S1), including *L3MBTL4 *and the potential TSG *EPB41L3/DAL1 *[28]. This suggests that this 18p11 region probably hosts multiple TSGs.

### Fourteen new 18p TSG candidates

To identify these other potential 18p11 TSGs we compared the degree of CNA-driven RNA downregulation in the 307 samples by analyzing the genes common to the genome and expression platforms (aCGH Agilent Technologies and Affymetrix) and retained after filtering based on the expression level. Sixty-three of the 64 were common. The gene expression of 14 genes (*YES1*, *SMCHD1*, *LPIN2*, *MRCL3*, *MRCL2*, *ZFP161*, *RALBP1*, *PPP4R1*, *NAPG*, *AFG3L2*, *SPIRE1*, *CEP76*, *PTPN2 *and *SEH1L) *was downregulated in relation with their copy number losses (p < 0.05) (Additional file 1, Table S6). We noted that *EPB41L3/DAL1 *and *L3MBTL4 *were not retained. The lowest expression of *EPB41L3 *in breast tumors was associated with the presence of lymph node metastases (p < 0.05), correlated with basal and luminal B molecular subtypes (p < 0.01) and showed clinical impact (Additional file 1, Table S7).

Among the 14 genes, the downregulated expressions of *ZFP161, PPP4R1 *and *YES1 *in breast tumors were correlated with the luminal B molecular subtype (p < 0.01). Interestingly, the downregulated expression of *ZFP161 *was correlated with a poor clinical outcome (5-years MFS, p = 1.7.10^-3^; 5-years OS, p = 1.6.10^-3^) (Additional file 1, Table S8).

## Discussion

### *L3MBTL4 *gene alterations

Few works have reported the presence of TSGs on 18p for breast cancer [1,2] and only *EPB41L3*/*DAL1 *[28] has been identified as potential TSG involved in this neoplasm thus far. Our aCGH results provide a detailed view of the alterations of chromosome arm 18p in breast tumors. Losses at 18p11.31 occurred in 25% of tumors and the smallest common region of deletion targeted the *L3MBTL4 *locus and was also the site of breakages. The karyotype of the MDA-MB-453 cell line displays a der(18)t(7;18) [29]. The *L3MBTL4 *breakpoint suggests involvement of this gene in this alteration. *L3MBTL4 *loss was associated with high grade and with lymph node metastasis. In agreement, an increased risk of relapse in patients with high risk breast cancer has been associated with 18p loss [30]. *L3MBTL4 *loss was associated with the luminal B subtype, which is characterized by a poor prognosis. Interestingly, *L3MBTL4 *is centromeric and in close proximity to the loss of heterozygosity (LOH) region spanning *EPB41L3 *previously reported in non-small cell lung carcinomas [1], breast carcinomas [3], and meningiomas [31].

*L3MBTL4 *was targeted by point mutations in few cases. These mutations clustered in the vicinity of the MBT or SAM motifs. A study of L3MBTL1 has shown that a point mutation in the second MBT repeat motif affected the binding to H1K26me and H4K20me [32]. We surmise that the nonsense mutation in the third MBT motif of L3MBTL4 may have similar functional implication.

In three tumors and in the BT-483 cell line *L3MBTL4 *mutations were associated with the loss of the other allele, suggesting a double-hit mechanism leading to complete loss-of-function of L3MBTL4 in these samples. We did not find homozygous mutations or deletions of *L3MBTL4*. However, we cannot exclude that in addition to these alterations, total inactivation could result from promoter hypermethylation of the other allele. The study of mRNA expression tended to confirm this.

### *L3MBTL4 *loss of expression

Expression of *L3MBTL4 *level was decreased at least two-fold in 73% of breast cancer samples, particularly in tumors deleted at the *L3MBTL4 *locus since decreased expression correlated with genomic alteration. All the deleted tumors showed absence or decreased *L3MBTL4 *expression. Few cases displayed mRNA downregulation in the absence of loss suggesting that other mechanisms such as epigenetic repression play a role in the downregulation of *L3MBTL4 *in breast tumors.

*L3MBTL4 *downregulated expression was not found only in luminal B tumors but also in luminal A, luminal B, ERBB2 and normal-like cases. Only the basal subtype presented a normal expression of the gene, independently of loss. This suggests that L3MBTL4 does not play a TSG role in this subtype of breast cancer. Inactivation of other TSGs may explain 18p losses in basal breast cancers.

### *L3MBTL4 *as a tumor suppressor gene

Our observations are in agreement with a previous study that suggested the presence of TSGs on 18p with a role in the genesis of breast cancer [33]. There are several reasons to believe that *L3MBTL4 *is a good candidate TSG. First, it lies within the region of chromosome 18 that is frequently deleted in breast cancers; *L3MBTL4 *is also targeted by mutations and breakages and is dowregulated in tumors. Second, *L3MBTL4 *gene is a human homolog of *Drosophila l(3)mbt*, which functions as a TSG in fly. The loss-of-function generated by mutation of *l(3)mbt *causes brain tumors in *Drosophila *[4,5]. Third, *L3MBTL4 *has three paralogs that are suspected to play a role in the etiology of certain types of cancer [7]. *L3MBTL1*, a known transcriptional repressor [34], has been proposed as a TSG gene in myeloid malignancies associated with 20q deletion [8,35,36]. A recent study reported focal hemi- and homozygous deletions of *L3MBTL2 *and *L3MBTL3 *in medulloblastoma [9].

The four human L3MBTL proteins have MBT domains involved in transcriptional repression and chromatin remodeling. The MBT domain was originally identified in the *Drosophila *l(3)mbt protein [5] and binds methyl-lysine residues [37,38], particularly and strongly H3K9me and H4K20me [39]. The transcriptional repressor L3MBTL1 requires its three MBT domains for compacting chromatin and silencing [32]. Although the exact biochemical properties and cellular functions of L3MBTL4 are unclear to date, the presence of MBT domains in L3MBTL4 suggests that it interacts with chromatin [40], may potentially bind methylated histone and thus play a role in transcriptional regulation of stem cell genes, oncogenes and tumor suppressors. Loss of this regulator may thus affect several breast cancer genes. Some of these genes may be part of the E2F/RB pathway. Indeed, the E2F/RB pathway is altered in luminal B cancers and L3MBTL proteins are known to regulate this pathway [7].

Taken together, our results suggest that aberrations targeting *L3MBTL4 *could confer to cancer cells specific advantages but do not exclude the role of other potential 18p candidates.

### Other 18p TSG candidates

One of the potential 18p TSGs might be *EPB41L3/DAL1 *[28], which is just telomeric to *L3MBTL4*, between the D18S59 and D18S452 markers. However, its expression was not correlated with that of *L3MBTL4*. We also identified 14 TSG candidates whose gene expression was downregulated in relation with their copy number losses. We noted that *EPB41L3/DAL1 *and *L3MBTL4 *were not included in the 14 suggesting that their downregulated gene expression is not only the consequence of their copy number loss but could result from other mechanisms of deregulation including epigenetic modifications. This is in agreement with study showing that the hypermethylation of *EPB41L3/DAL1 *was associated with its downregulation in lung cancer [41]. Our *EPB41L3/DAL1 *expression data showed that its downregulation could be associated with an increased risk of relapse in patients with high risk breast cancer.

*EPB41L3/DAL1 *undergoes allelic losses in various cancers and in a significant proportion of ductal carcinomas *in situ *of the breast. The EPB41L3/DAL1 protein suppresses the growth of MCF7 breast cancer cells and increases attachment of these cells to a variety of extracellular matrices [28]. Modulation of post-translational methylation may be an important mechanism through which EPB41L3/DAL1 affects tumor cell growth [42]. EPB41L3/DAL1 plays a critical role in the suppression of lung tumor formation and metastasis [43]. However, the role of *EPB41L3/DAL1 *as a TSG has yet to be validated *in vivo*. EPB41L3/DAL1 deficient mice are healthy and do not develop spontaneous tumors [44]. Mutational screening failed to identify inactivating mutation of the *EPB41L3/DAL1 *gene [33].

Among the 14 other TSG candidates, except PTPN2/TCTP none has been so far associated with cancer. *PTPN2*/*TCPTP *codes for the T-cell protein tyrosine phosphatase (TCPTP) and is an important negative regulator of SFK, JAK1 and STAT3 signaling during the cell cycle [45]. TCPTP suppresses the tumorigenicity of glioblastoma cells expressing a mutant epidermal growth factor receptor [46]. *CEP76 *encodes a centrosomal protein controling centrosome duplication during cell division. Abnormal centrosome duplication contributes to mitotic failure, genome instability, aneuploidy, and cancer. Depletion of CEP76 drives the accumulation of centrosome intermediates in certain types of cancer cells [47]. Only the downregulated expressions of *ZFP161, PPP4R1 *and *YES1 *were correlated with the luminal B subtype suggesting their potential involvement in the genesis of a particularly aggressive form of breast cancer with 18p loss. The downregulated expression of *ZFP161 *in breast tumors was correlated with a poor clinical evolution. *ZFP161/ZF5 *encodes a ubiquitously-expressed protein originally identified by its ability to bind and repress the murine *Myc *promoter [48,49]. The protein contains an N-terminal POZ domain, which recruits cofactors to modulate transcription [50]. ZFP161/ZF5 mediates both transcriptional activation and repression of cellular and viral promoters [48,50,51]. ZFP161 may compete with MYC-induced transcription [52].

*PPP4R1 *encodes the regulatory subunit of a ~125-kDa protein phosphatase. PPP4R1 interacts with PPP4C [53,54] which is implicated in the regulation of histone acetylation, DNA damage checkpoint signaling, NFκB activation, and microtubule organization at centrosomes [55-59]. *YES1 *encodes a SRC-family kinase [60] and its tyrosine kinase activity has been shown to be elevated in colonic adenomas compared to its activity in adjacent normal mucosa [61]. A number of studies have linked increased expression of YES in cancer with increased cell motility and tumor invasion [62,63]. It is then surprising to find *YES1 *among the 18p TSG candidates. The downregulation of *YES1 *could be the consequence of its loss as a simple passenger of a larger region lost within 18p.

## Conclusion

We have delineated a region of frequent loss in breast cancer on chromosome arm 18p. We have identified *L3MBTL4 *as the gene targeted by these losses. *L3MBTL4 *is also targeted by mutations and breakages. *L3MBTL4 *mRNA expression is low in non-basal breast tumors and in particular in tumors with loss of the gene. Alteration of *L3MBTL4*, coding for a regulator of epigenetic marks, is well in line with recent advances in cancer research [64,65]. We have also pointed to other 18p TSG candidates including *ZFP161, CEP76, PPP4R1 *and *PTPN2 *whose involvement might explain the aggressive phenotype of breast tumors with 18p loss.

## Competing interests

The authors declare that they have no competing interests.

## Authors' contributions

LAK and NC did sequence and qRT-PCR analyses, JA, IB, FS and AF aCGH experiments and analysis, PF and FB gene expression analysis, PV and SM supervised the study, DB and MC designed and supervised the study, LAK and MC wrote the manuscript. All authors read and approved the final manuscript.

## Supplementary Material

Additional file 1 **Supplementary Tables S1-S8**. **Table S1**. Clinical and histological features of 307 breast tumor cases. Table S2. Primers used for *L3MBTL4 *sequence determination. Table S3. Association of genes with copy number alterations and *L3MBTL4 *genomic loss. Table S4A. The 77 tumors with *L3MBTL4 *loss and mutation. Table S4B. Correlations between clinical features of 305 BC and the loss of whole chromosome 18p. Table S5. Correlations between clinical features of 229 BC and *L3MBTL4 *gene expression. Table S6 - Other potential 18p TSG candidates. Table S7. Correlations between clinical features of 260 BC and *EPB41L3 *gene expression. Table S8. Correlations between clinical features of 260 BC and down regulated ZFP161 gene expression. Click here for file

Additional file 2 **Supplementary figure**. **Figure S1**. The 18p is targeted by losses in breast cancer. On the top, combining the CNA frequency and gene copy number alterations level, the GISTIC algorithm plotted the score index observed in genomic profiles of 307 breast tumors as a function of chromosome 18 locations. The chromosome arm 18p was targeted by losses in more than 20% of tumor samples. The dotted line indicates the threshold of significance for the score. At the bottom, the figure shows only an 18p11.21-p11.32 region of 12.79 Mb including 64 loci as significant 18p losses (p < 10^-5^). *L3MBTL4 *and *EPB41L3 *are contained within this region. Click here for file

## References

[B1] Tran Y Benbatoul K Gorse K Rempel S Futreal A Green M Newsham I Novel regions of allelic deletion on chromosome 18p in tumors of the lung, brain and breast Oncogene 1998 17 3499 3505 10.1038/sj.onc.1202258 10030674

[B2] Osborne RJ Hamshere MG A Genome-wide Map Showing Common Regions of Loss of Heterozygosity/AllelicImbalance in Breast Cancer Cancer Res 2000 60 3706 3712 10919637

[B3] Kittiniyom K Gorse KM Dalbegue F Lichy JH Taubenberger JK Newsham IF Allelic loss on chromosome band 18p11.3 occurs early and reveals heterogeneity in breast cancer progression Breast Cancer Res 2001 3 192 198 10.1186/bcr294 11305954 PMC30703

[B4] Gateff E Löffler T Wismar J A temperature-sensitive brain tumor suppressor mutation of Drosophila melanogaster: developmental studies and molecular localization of the gene Mech Dev 1993 41 15 31 10.1016/0925-4773(93)90052-Y 8507589

[B5] Wismar J Löffler T Habtemichael N Vef O Geissen M Zirwes R Altmeyer W Sass H Gateff E The Drosophila melanogaster tumor suppressor gene lethal(3)malignant brain tumor encodes a proline-rich protein with a novel zinc finger Mech Dev 1995 53 141 154 10.1016/0925-4773(95)00431-9 8555106

[B6] Wismar J Molecular characterization of h-l(3)mbt-like: a new member of the human mbt family FEBS Lett 2001 507 119 121 10.1016/S0014-5793(01)02959-3 11682070

[B7] Bonasio R Lecona E Reinberg D MBT domain proteins in development and disease Semin Cel Dev Biol 2010 21 221 230 10.1016/j.semcdb.2009.09.010 PMC3772645 19778625

[B8] Li J Bench AJ Piltz S Vassiliou G Baxter EJ Ferguson-Smith AC Green AR L3mbtl, the mouse orthologue of the imprinted L3MBTL, displays a complex pattern of alternative splicing and escapes genomic imprinting Genomics 2005 86 489 494 10.1016/j.ygeno.2005.06.012 16081246

[B9] Northcott PA Nakahara Y Wu X Feuk L Ellison DW Croul S Mack S Kongkham PN Peacock J Dubuc A Ra YS Zilberberg K McLeod J Scherer SW Sunil Rao J Eberhart CG Grajkowska W Gillespie Y Lach B Grundy R Pollack IF Hamilton RL Van Meter T Carlotti CG Boop F Bigner D Gilbertson RJ Rutka JT Taylor MD Multiple recurrent genetic events converge on control of histone lysine methylation in medulloblastoma Nat Genet 2009 41 465 472 10.1038/ng.336 19270706 PMC4454371

[B10] Tomlinson GE Chen TT Stastny VA Virmani AK Spillman MA Tonk V Blum JL Schneider NR Wistuba II Shay JW Minna JD Gazdar AF Characterization of a breast cancer cell line derived from a germ-line BRCA1 mutation carrier Cancer Res 1998 58 3237 3242 9699648

[B11] Hackett AJ Smith HS Springer EL Owens RB Nelson-Rees WA Riggs JL Gardner MB Two syngeneic cell lines from human breast tissue: the aneuploid mammary epithelial (Hs578T) and the diploid myoepithelial (Hs578Bst) cell lines J Natl Cancer Inst 1977 58 1795 1806 864756 10.1093/jnci/58.6.1795

[B12] Möbus VJ Moll R Gerharz CD Kieback DG Merk O Runnebaum IB Linner S Dreher L Grill HJ Kreienberg R Differential characteristics of two new tumorigenic cell lines of human breast carcinoma origin Int J Cancer 1998 77 415 423 10.1002/(SICI)1097-0215(19980729)77:3<415::AID-IJC18>3.0.CO;2-6 9663605

[B13] The University of Michigan Human Breast Cancer Cell Lines (SUM-LINES) http://www.cancer.med.umich.edu/breast_cell/Production/index.html

[B14] Gioanni J Le François D Zanghellini E Mazeau C Ettore F Lambert JC Schneider M Dutrillaux B Establishment and characterisation of a new tumorigenic cell line with a normal karyotype derived from a human breast adenocarcinoma Br J Cancer 1990 62 8 13 2390488 10.1038/bjc.1990.219 PMC1971752

[B15] Theillet C Adelaïde J Louason G Bonnet-Dorion F Jacquemier J Adnane J Longy M Katsaros D Sismondi P Gaudray P Birnbaum D FGFRI and PLAT genes and DNA amplification at 8p12 in breast and ovarian cancers Genes Chromosomes Cancer 1993 7 219 226 10.1002/gcc.2870070407 7692948

[B16] Adélaïde J Finetti P Bekhouche I Repellini L Geneix J Sircoulomb F Charafe-Jauffret E Cervera N Desplans J Parzy D Schoenmakers E Viens P Jacquemier J Birnbaum D Bertucci F Chaffanet M Integrated profiling of basal and luminal breast cancers Cancer Res 2007 67 11565 11575 10.1158/0008-5472.CAN-07-2536 18089785

[B17] Olshen AB Venkatraman ES Lucito R Wigler M Circular binary segmentation for the analysis of array-based DNA copy number data Biostatistics 2004 5 557 572 10.1093/biostatistics/kxh008 15475419

[B18] Irizarry RA Hobbs B Collin F Beazer-Barclay YD Antonellis KJ Scherf U Speed TP Exploration, normalization, and summaries of high density oligonucleotide array probe level data Biostatistics 2003 4 249 264 10.1093/biostatistics/4.2.249 12925520

[B19] McCarroll SA Kuruvilla FG Korn JM Cawley S Nemesh J Wysoker A Shapero MH de Bakker PI Maller JB Kirby A Elliott AL Parkin M Hubbell E Webster T Mei R Veitch J Collins PJ Handsaker R Lincoln S Nizzari M Blume J Jones KW Rava R Daly MJ Gabriel SB Altshuler D Integrated detection and population-genetic analysis of SNPs and copy number variation Nat Genet 2008 40 1166 1174 10.1038/ng.238 18776908

[B20] Iafrate AJ Feuk L Rivera MN Listewnik ML Donahoe PK Qi Y Scherer SW Lee C Detection of large-scale variation in the human genome Nat Genet 2004 36 949 951 10.1038/ng1416 15286789

[B21] Beroukhim R Getz G Nghiemphu L Barretina J Hsueh T Linhart D Vivanco I Lee JC Huang JH Alexander S Du J Kau T Thomas RK Shah K Soto H Perner S Prensner J Debiasi RM Demichelis F Hatton C Rubin MA Garraway LA Nelson SF Liau L Mischel PS Cloughesy TF Meyerson M Golub TA Lander ES Mellinghoff IK Sellers WR Assessing the significance of chromosomal aberrations in cancer: methodology and application to glioma Proc Natl Acad Sci USA 2007 104 20007 200012 10.1073/pnas.0710052104 18077431 PMC2148413

[B22] Bertucci Finetti P Cervera N Charafe-Jauffret E Mamessier E Adélaïde J Debono S Houvenaeghel G Maraninchi D Viens P Charpin C Jacquemier J Birnbaum D Gene expression profiling shows medullary breast cancer is a subgroup of basal breast cancers Cancer Res 2006 66 4636 4644 10.1158/0008-5472.CAN-06-0031 16651414

[B23] Hu Z Fan C Oh DS Marron JS He X Qaqish BF Livasy C Carey LA Reynolds E Dressler L Nobel A Parker J Ewend MG Sawyer LR Wu J Liu Y Nanda R Tretiakova M Ruiz Orrico A Dreher D Palazzo JP Perreard L Nelson E Mone M Hansen H Mullins M Quackenbush JF Ellis MJ Olopade OI Bernard PS Perou CM The molecular portraits of breast tumors are conserved across microarray platforms BMC Genomics 2006 7 96 10.1186/1471-2164-7-96 16643655 PMC1468408

[B24] Benito M Parker J Du Q Wu J Xiang D Perou CM Marron JS Adjustment of systematic microarray data biases Bioinformatics 2004 20 105 114 10.1093/bioinformatics/btg385 14693816

[B25] Benjamini Y Hochberg Y Controlling the false discovery rate - a practical and powerful approach to multiple testing J Roy Stat Soc B Met 1995 57 289 300

[B26] Snijders AM Pinkel D Albertson DG Current status and future prospects of array-based comparative genomic hybridisation Brief Funct Genomic Proteomic 2003 2 37 45 10.1093/bfgp/2.1.37 15239942

[B27] NCBI db SNP build 131 for human http://www.ncbi.nlm.nih.gov/SNP

[B28] Charboneau AL Singh V Yu T Newsham IF Suppression of growth and increased cellular attachment after expression of DAL-1 in MCF-7 breast Cancer Cells Int J Cancer 2002 100 181 188 10.1002/ijc.10470 12115567

[B29] Popovici C Basset C Bertucci F Orsetti B Adélaide J Mozziconacci MJ Conte N Murati A Ginestier C Charafe-Jauffret E Ethier SP Lafage-Pochitaloff M Theillet C Birnbaum D Chaffanet M Reciprocal translocations in breast tumor cell lines: cloning of a t(3;20) that targets the FHIT gene Genes Chromosomes Cancer 2003 35 204 218 10.1002/gcc.10107 12353263

[B30] Climent J Martinez-Climent JA Blesa D Garcia-Barchino MJ Saez R Sánchez-Izquierdo D Azagra P Lluch A Garcia-Conde J Genomic loss of 18p predicts an adverse clinical outcome in patients with high-risk breast cancer Clin Cancer Res 2002 8 3863 3869 12473601

[B31] Gutmann DH Donahoe J Perry A Lemke N Gorse K Kittiniyom K Rempel SA Gutierrez JA Newsham IF Loss of DAL-1, a protein 4.1-related tumor suppressor, is an important early event in the pathogenesis of meningiomas Hum Mol Genet 2000 9 1495 1500 10.1093/hmg/9.10.1495 10888600

[B32] Trojer P Li G Sims RJ Vaquero A Kalakonda N Boccuni P Lee D Erdjument-Bromage H Tempst P Nimer SD Wang YH Reinberg D L3MBTL1, a histone-methylation-dependent chromatin lock Cell 2007 129 915 928 10.1016/j.cell.2007.03.048 17540172

[B33] Kittiniyom K Mastronardi M Roemer M Wells WA Greenberg ER Titus-Ernstoff L Newsham IF Allele-specific loss of heterozygosity at the DAL-1/4.1B (EPB41L3) tumor-suppressor gene locus in the absence of mutation Genes Chromosomes Cancer 2004 40 190 203 10.1002/gcc.20034 15138999

[B34] Boccuni P MacGrogan D Scandura JM Nimer SD The human L(3)MBT polycomb group protein is a transcriptional repressor and interacts physically and functionally with TEL (ETV6) J Biol Chem 2003 278 15412 15420 10.1074/jbc.M300592200 12588862

[B35] Bench AJ Li J Huntly BJ Delabesse E Fourouclas N Hunt AR Deloukas P Green AR Characterization of the imprinted polycomb gene L3MBTL, a candidate 20q tumour suppressor gene, in patients with myeloid malignancies Br J Haematol 2004 127 509 518 10.1111/j.1365-2141.2004.05278.x 15566354

[B36] MacGrogan D Kalakonda N Alvarez S Scandura JM Boccuni P Johansson B Nimer SD Structural integrity and expression of the *L3MBTL *gene in normal and malignant hematopoietic cells Genes Chromosomes Cancer 2004 41 203 213 10.1002/gcc.20087 15334543

[B37] Kim J Daniel J Espejo A Lake A Krishna M Xia L Zhang Y Bedford MT Tudor, MBT and chromo domains gauge the degree of lysine methylation EMBO Rep 2006 7 397 403 16415788 10.1038/sj.embor.7400625 PMC1456902

[B38] Min J Allali-Hassani A Nady N Qi C Ouyang H Liu Y MacKenzie F Vedadi M Arrowsmith CH L3MBTL1 recognition of mono- and dimethylated histones Nat Struct Mol Biol 2007 14 1229 1230 10.1038/nsmb1340 18026117

[B39] Klymenko T Papp B Fischle W Köcher T Schelder M Fritsch C Wild B Wilm M Müller J A Polycomb group protein complex with sequence-specific DNA-binding and selective methyl-lysine-binding activities Genes Dev 2006 20 1110 1122 10.1101/gad.377406 16618800 PMC1472471

[B40] Guo Y Nady N Qi C Allali-Hassani A Zhu H Pan P Adams-Cioaba MA Amaya MF Dong A Vedadi M Schapira M Read RJ Arrowsmith CH Min J Methylation-state-specific recognition of histones by the MBT repeat protein L3MBTL2 Nucleic Acids Res 2009 37 2204 2210 10.1093/nar/gkp086 19233876 PMC2673432

[B41] Kikuchi S Yamada D Fukami T Masuda M Sakurai-Yageta M Williams YN Maruyama T Asamura H Matsuno Y Onizuka M Murakami Y Promoter methylation of DAL-1/4.1B predicts poor prognosis in non-small cell lung cancer Clin Cancer Res 2005 11 2954 2961 10.1158/1078-0432.CCR-04-2206 15837747

[B42] Singh V Miranda TB Jiang W Frankel A Roemer ME Robb VA Gutmann DH Herschman HR Clarke S Newsham IF DAL-1/4.1B tumor suppressor interacts with protein arginine N-methyltransferase 3 (PRMT3) and inhibits its ability to methylate substrates in vitro and in vivo Oncogene 2004 23 7761 7771 10.1038/sj.onc.1208057 15334060

[B43] Yageta M Kuramochi M Masuda M Fukami T Fukuhara H Maruyama T Shibuya M Murakami Y Direct association of TSLC1 and DAL-1, two distinct tumor suppressor proteins in lung cancer Cancer Res 2002 62 5129 5133 12234973

[B44] Yi C McCarty JH Troutman SA Eckman MS Bronson RT Kissil JL Loss of the putative tumor suppressor band 4.1B/Dal1 gene is dispensable for normal development and does not predispose to cancer Mol Cell Biol 2005 25 10052 10059 10.1128/MCB.25.22.10052-10059.2005 16260618 PMC1280276

[B45] Ibarra-Sanchez M Simoncic PD Nestel FR Duplay P Lapp WS Tremblay ML The T-cellprotein tyrosine phosphatase Semin Immunol 2000 12 379 386 10.1006/smim.2000.0220 10995584

[B46] Klingler-Hoffmann M Fodero-Tavoletti MT Mishima K Narita Y Cavenee WK Furnari FB Huang HJ Tiganis T The protein tyrosine phosphatase TCPTP suppresses the tumorigenicity of glioblastoma cells expressing a mutant epidermal growth factor receptor J Biol Chem 2001 276 46313 46318 10.1074/jbc.M106571200 11514572

[B47] Tsang WY Spektor A Vijayakumar S Bista BR Li J Sanchez I Duensing S Dynlacht BD Cep76, a centrosomal protein that specifically restrains centriole reduplication Dev Cell 2009 16 649 660 10.1016/j.devcel.2009.03.004 19460342 PMC4062978

[B48] Numoto M Niwa O Kaplan J Wong KK Merrell K Kamiya K Yanagihara K Calame K Transcriptional repressor ZF5 identifies a new conserved domain in zinc finger proteins Nucleic Acids Res 1993 21 3767 3775 10.1093/nar/21.16.3767 8367294 PMC309887

[B49] Sobek-Klocke I Disqué-Kochem C Ronsiek M Klocke R Jockusch H Breuning A Ponstingl H Rojas K Overhauser J Eichenlaub-Ritter U The human gene ZFP161 on 18p11.21-pter encodes a putative c-myc repressor and is homologous to murine Zfp161 (Chr 17) and Zfp161-rs1 (X Chr) Genomics 1997 43 156 64 10.1006/geno.1997.4784 9244432

[B50] Kaplan J Calame K The ZiN/POZ domain of ZF5 is required for both transcriptional activation and repression Nucleic Acids Res 1997 25 1108 1116 10.1093/nar/25.6.1108 9092617 PMC146578

[B51] Yokoro K Yanagidani A Obata T Yamamoto S Numoto M Genomic cloning and characterization of the mouse POZ/zinc-finger protein ZF5 Biochem Biophys Res Commun 1998 246 668 674 10.1006/bbrc.1998.8675 9618270

[B52] Reymann S Borlak J Transcription profiling of lung adenocarcinomas of c-myc-transgenic mice: Identification of the c-myc regulatory gene network BMC Syst Biol 2008 2 46 10.1186/1752-0509-2-46 18498649 PMC2430022

[B53] Kloeker S Wadzinski BE Purification and identification of a novel subunit of protein serine/threonine phosphatase 4 J Biol Chem 1999 274 5339 5347 10.1074/jbc.274.9.5339 10026142

[B54] Wada T Miyata T Inagi R Nangaku M Wagatsuma M Suzuki D Wadzinski BE Okubo K Kurokawa K Cloning and characterization of a novel subunit of protein serine/threonine phosphatase 4 from mesangial cells J Am Soc Nephrol 2001 12 2601 2608 11729228 10.1681/ASN.V12122601

[B55] Zhou G Mihindukulasuriya KA MacCorkle-Chosnek RA Van Hooser A Hu MC Brinkley BR Tan TH Protein phosphatase 4 is involved in tumor necrosis factor-alpha-induced activation of c-Jun N-terminal kinase J Biol Chem 2002 277 6391 6398 10.1074/jbc.M107014200 11698396

[B56] Cohen PT Philp A Vázquez-Martin C Protein phosphatase 4--from obscurity to vital functions FEBS Lett 2005 579 3278 3286 10.1016/j.febslet.2005.04.070 15913612

[B57] Zhang X Ozawa Y Lee H Wen YD Tan TH Wadzinski BE Seto E Histone deacetylase 3 (HDAC3) activity is regulated by interaction with protein serine/threonine phosphatase 4 Genes Dev 2005 19 827 839 10.1101/gad.1286005 15805470 PMC1074320

[B58] Chowdhury D Xu X Zhong X Ahmed F Zhong J Liao J Dykxhoorn DM Weinstock DM Pfeifer GP Lieberman JA PP4-phosphatase complex dephosphorylates gamma-H2AX generated during DNA replication Mol Cell 2008 31 33 46 10.1016/j.molcel.2008.05.016 18614045 PMC3242369

[B59] Nakada S Chen GI Gingras A-C Durocher D PP4 is a gamma H2AX phosphatase required for recovery from the DNA damage checkpoint EMBO Rep 2008 9 1019 1026 Erratum in *EMBO Rep*, 2008, 9:1251. 10.1038/embor.2008.162 18758438 PMC2527856

[B60] Thomas SM Brugge JS Cellular functions regulated by Src family kinases Annu Rev Cell Dev Biol 1997 13 513 609 10.1146/annurev.cellbio.13.1.513 9442882

[B61] Pena SV Melhem MF Meisler AI Cartwright CA Elevated c-yes tyrosine kinase activity in premalignant lesions of the colon Gastroenterology 1995 108 117 124 10.1016/0016-5085(95)90015-2 7806032

[B62] Barraclough J Hodgkinson C Hogg A Dive C Welman A Increases in c-Yes expression level and activity promote motility but not proliferation of human colorectal carcinoma cells Neoplasia 2007 9 745 754 10.1593/neo.07442 17898870 PMC1993859

[B63] Kleber S Sancho-Martinez I Wiestler B Beisel A Gieffers C Hill O Thiemann M Mueller W Sykora J Kuhn A Schreglmann N Letellier E Zuliani C Klussmann S Teodorczyk M Gröne HJ Ganten TM Sültmann H Tüttenberg J von Deimling A Regnier-Vigouroux A Herold-Mende C Martin-Villalba A Yes and PI3K bind CD95 to signal invasion of glioblastoma Cancer Cell 2008 13 235 248 10.1016/j.ccr.2008.02.003 18328427

[B64] Van Haaften G Dalgliesh GL Davies H Chen L Bignell G Greenman C Edkins S Hardy C O'Meara S Teague J Butler A Hinton J Latimer C Andrews J Barthorpe S Beare D Buck G Campbell PJ Cole J Forbes S Jia M Jones D Kok CY Leroy C Lin ML McBride DJ Maddison M Maquire S McLay K Menzies A Mironenko T Mulderrig L Mudie L Pleasance E Shepherd R Smith R Stebbings L Stephens P Tang G Tarpey PS Turner R Turrell K Varian J West S Widaa S Wray P Collins VP Ichimura K Law S Wong J Yuen ST Leung SY Tonon G DePinho RA Tai YT Anderson KC Kahnoski RJ Massie A Khoo SK Teh BT Stratton MR Futreal PA Somatic mutations of the histone H3K27 demethylase gene UTX in human cancer Nat Genet 2009 41 521 523 10.1038/ng.349 19330029 PMC2873835

[B65] Figueroa ME Lugthart S Li Y Erpelinck-Verschueren C Deng X Christos PJ Schifano E Booth J van Putten W Skrabanek L Campagne F Mazumdar M Greally JM Valk PJ Löwenberg B Delwel R Melnick A DNA methylation signatures identify biologically distinct subtypes in acute myeloid leukemia Cancer Cell 2010 17 13 27 10.1016/j.ccr.2009.11.020 20060365 PMC3008568

