# Supplementary figures and images for: Loss, mutation and deregulation of L3MBTL4 in breast cancers
Source: Mol Cancer. 2010 Aug 10;9:213. doi: 10.1186/1476-4598-9-213 (PMC2933619; doi:10.1186/1476-4598-9-213)

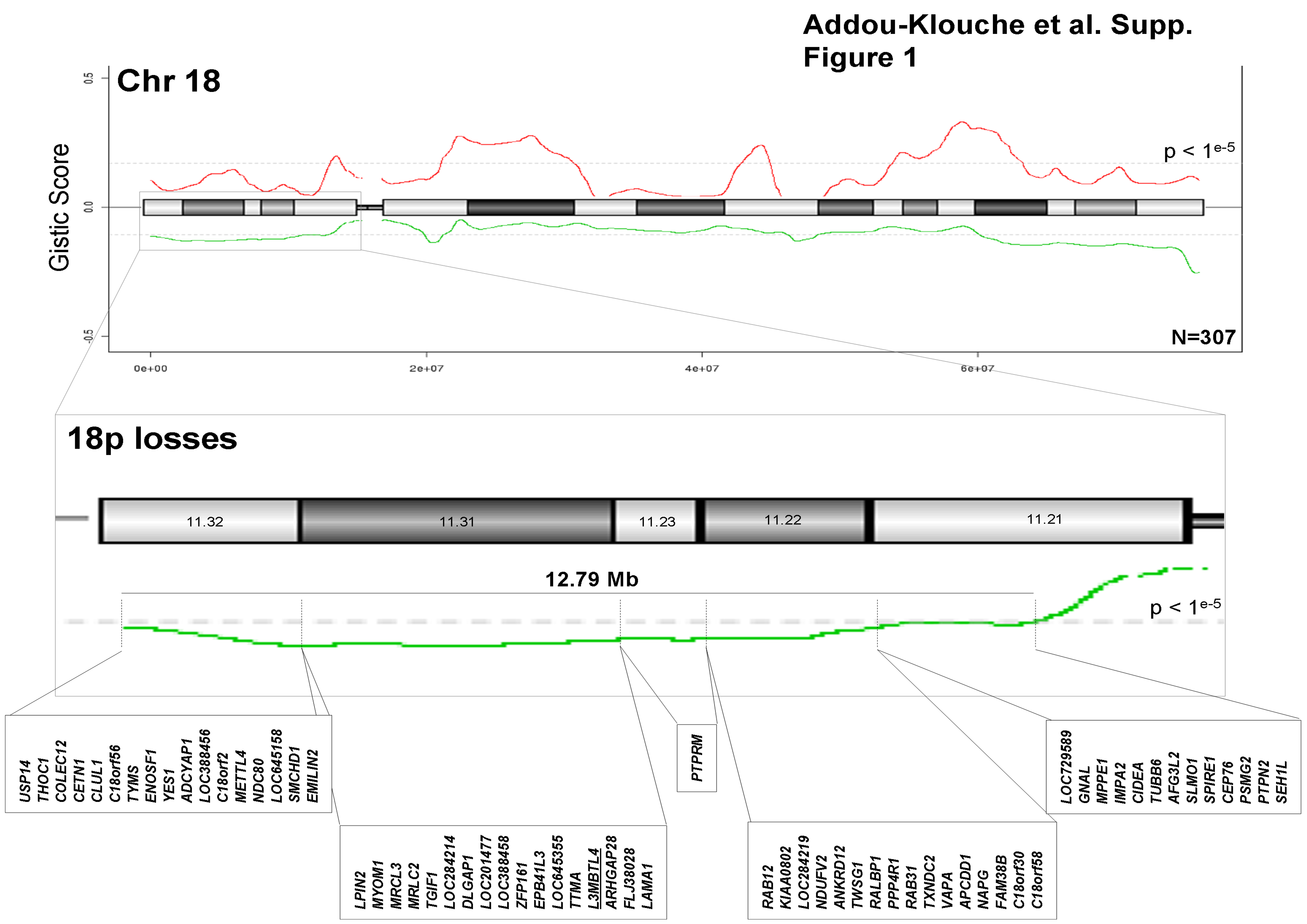

Supplement: Additional file 2 — Supplementary figure. Figure S1. The 18p is targeted by losses in breast cancer. On the top, combining the CNA frequency and gene copy number alterations level, the GISTIC algorithm plotted the score index observed in genomic profiles of 307 breast tumors as a function of chromosome 18 locations. The chromosome arm 18p was targeted by losses in more than 20% of tumor samples. The dotted line indicates the threshold of significance for the score. At the bottom, the figure shows only an 18p11.21-p11.32 region of 12.79 Mb including 64 loci as significant 18p losses (p < 10-5). L3MBTL4 and EPB41L3 are contained within this region. [file 1476-4598-9-213-S2.TIFF]
